# EndophilinAs regulate endosomal sorting of BDNF-TrkB to mediate survival signaling in hippocampal neurons

**DOI:** 10.1038/s41598-017-02202-4

**Published:** 2017-05-19

**Authors:** Katja Burk, John D. Murdoch, Siona Freytag, Melanie Koenig, Vinita Bharat, Ronja Markworth, Susanne Burkhardt, Andre Fischer, Camin Dean

**Affiliations:** 1 European Neuroscience Institute, ENI-G, 37077 Goettingen, Germany; 2 German Center for Neurodegenerative Diseases (DZNE), University Medical Center Goettingen, 37075 Goettingen, Germany

## Abstract

The sorting of activated receptors into distinct endosomal compartments is essential to activate specific signaling cascades and cellular events including growth and survival. However, the proteins involved in this sorting are not well understood. We discovered a novel role of EndophilinAs in sorting of activated BDNF-TrkB receptors into late endosomal compartments. Mice lacking all three EndophilinAs accumulate Rab7-positive late endosomes. Moreover, EndophilinAs are differentially localized to, co-traffic with, and tubulate, distinct endosomal compartments: In response to BDNF, EndophilinA2 is recruited to both early and late endosomes, EndophilinA3 is recruited to Lamp1-positive late endosomes, and co-trafficks with Rab5 and Rab7 in both the presence and absence of BDNF, while EndophilinA1 colocalizes at lower levels with endosomes. The absence of all three EndophilinAs caused TrkB to accumulate in EEA1 and Rab7-positive endosomes, and impaired BDNF-TrkB-dependent survival signaling cascades. In addition, EndophilinA triple knockout neurons exhibited increased cell death which could not be rescued by exogenous BDNF, in a neurotrophin-dependent survival assay. Thus, EndophilinAs differentially regulate activated receptor sorting via distinct endosomal compartments to promote BDNF-dependent cell survival.

## Introduction

Endophilins were initially discovered through a screen for SH3 domain-containing proteins in a mouse embryonic cDNA library. Three EndophilinA genes were identified: EndophilinA1, EndophilinA2 and EndophilinA3^1^. All EndophilinAs are present in the brain: EndophilinA1 is brain-specific, EndophilinA2 is expressed in all tissues, and EndophilinA3 is expressed at high levels in brain and testes^2–4^. The role of EndophilinA has been well-described in clathrin-mediated endocytosis of synaptic vesicles: EndophilinA recruits synaptojanin1 (a PI(4,5)P_2_ phosphatase) to the neck of newly formed clathrin-coated endocytic vesicles, which promotes disassembly of the clathrin coat^5, 6^. More recently, EndophilinAs have also been reported to mediate endocytosis of activated receptors, including G-protein coupled receptors and receptor tyrosine kinases, via a fast clathrin-independent mechanism, where EndophilinA triple knockdown fibroblasts had deficits in endocytosis of activated over-expressed receptors^7^.

EndophilinAs contain BAR domains, which promote membrane tubulation^8, 9^, and SH3 domains, which recruit dynamin to mediate membrane scission^10^. Consistent with a role in bending membranes during endocytosis, EndophilinAs are present in plasma membrane and synaptic vesicle subcellular biochemical fractions^11^. But EndophilinAs are also present in the cytosol^12, 13^, and in light membrane fractions which include endosomes^14^.

Endosomal compartments act as sorting platforms to determine the fate of internalized receptors - sending them into recycling endosomes targeted to the plasma membrane, to late “signaling” endosomes destined for long-range trafficking to the soma, or to lysosomes for degradation. The sorting of activated receptors to different endosomal compartments is accomplished by the retromer complex, which sorts endocytosed receptors into tubular microdomains of early endosomes formed by the sorting nexins^15^. These tubules are then pinched off at the very tip by dynamin to form vesicles which are then routed via endosomal markers for recycling, long-range trafficking, or degradation^16^. The sorting nexins contain BAR domains, which promote membrane tubulation, and an SH3 domain, which recruits dynamin for scission. We hypothesized that EndophilinAs - which also contain BAR domains and SH3 domains - may be present on endosomal compartments to promote the sorting of activated receptors, in addition to their well-known role in endocytosis from the plasma membrane. Importantly, endosomal sorting regulates the signaling of activated receptors: Signaling molecules such as the small GTPases Ras and Rap are located on distinct endosomal compartments and the sorting of activated receptors into these compartments promotes their interaction with these signaling molecules^17, 18^.

BDNF-activated TrkB receptors have been reported to traffic in Rab7-positive “signaling endosomes” towards the cell soma^19^. In the soma, phosphorylated TrkB promotes cell survival by activating signaling cascades leading to transcription of survival genes or by inhibiting pro-apoptotic signals^1, 20–22^. However, the proteins involved in the endosomal sorting of TrkB in order to activate the correct signaling cascades are not well understood. Here we discovered a novel function of EndophilinAs, in addition to their well-described role in synaptic vesicle and receptor endocytosis: EndophilinAs regulate endosomal sorting of activated TrkB receptors and subsequent signaling cascades to promote cell survival.

## Results

### Lack of EndophilinAs causes accumulation of Rab7 late endosomes

**Figure 1:**
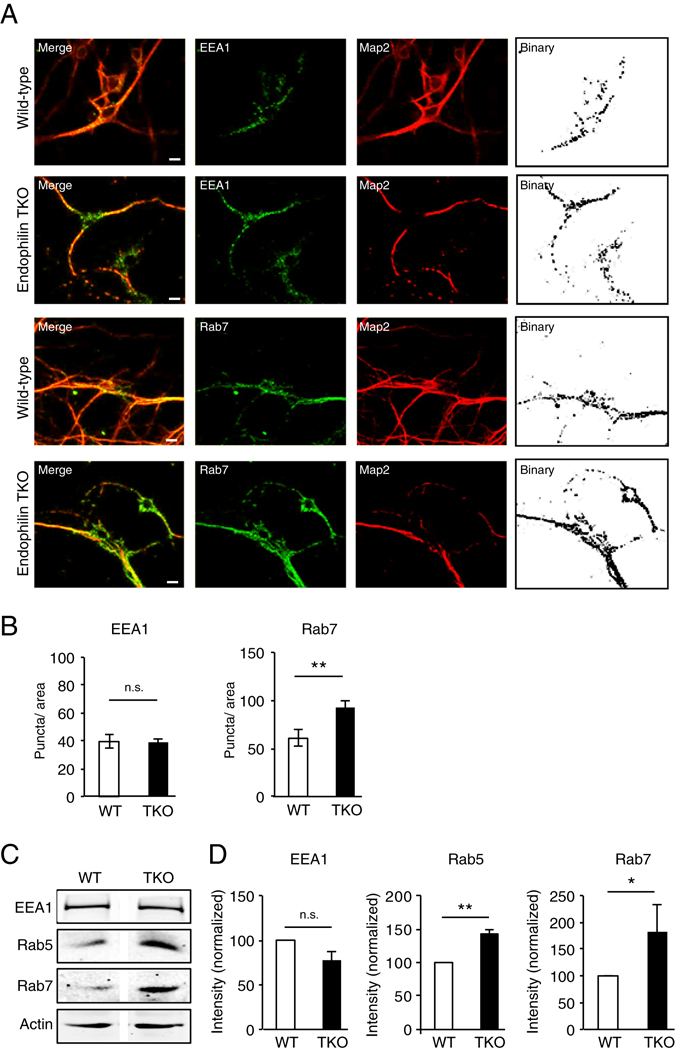
Rab7-positive endosomes accumulate in EndophilinA TKO hippocampal neurons. (**A**) Identification of endosomal structures in wild-type and EndophilinA TKO neurons using immunocytochemistry to detect endogenous levels of the endosomal markers EEA1, and Rab7. Representative images of WT and Endophilin TKO hippocampal neurons stained for EEA1 and Rab7 are shown. Map2 antibody was used to label neurons. Scale bar = 10 μm; (**B**) Quantification of endosomal marker signal for EEA1 or Rab7 (**C**) normalized to area. Significance determined by unpaired two-tailed Student’s t-test comparing each condition to control, n = 30 images per condition from 3 independent cell cultures; error = SEM, **p < 0.01. (**C**) Western blots showing levels of EEA1 and Rab7 in wild-type and EndophilinA TKO brain homogenates. (**D**) Quantitation of protein levels of EEA1 and Rab7 (F) in control and EndophilinA TKOs normalized to actin; n = 3 independent brain samples, error = SEM, *p < 0.05, **p < 0.01.

To determine if EndophilinAs have a role in endosomal sorting, we first investigated if early (EEA1-positive) or late (Rab7-positive) endosomes were altered in EndophilinA1-/-, A2-/- A3-/- triple knockout mice (henceforth TKO)^6^ compared to control wild-type mice using immunocytochemistry to label endogenous levels of EEA1 and Rab7 in hippocampal neurons (Fig. 1A). Binary images of EEA1 or Rab7 positive-immunolabelling were analysed for number of puncta per area. While we found no change in the number of EEA1-positive immunolabelled puncta per area, a significant increase in the number of Rab7 positive puncta appeared in EndophilinA TKO neurons compared to wild-type neurons (Fig. 1A,B). Interestingly, we also found more punctate Map2 staining and less Map2 in cell bodies of TKO neurons, compared to WT controls. To confirm the increase in Rab7 in EndophilinA TKOs, we performed Western blot analysis to test protein levels, which revealed a significant increase in Rab5 and Rab7, and unchanged levels of EEA1 in EndophilinA TKO brain homogenates (Fig. 1C,D; see Suppl. Fig. 1 for full blots). Thus, lack of all three EndophilinAs leads to the accumulation of Rab5 and Rab7-positive late endosomes, suggesting that EndophilinAs may localize to endosomes to promote their sorting.

### EndophilinA localizes to and co-traffics with endosomes

**Figure 2:**
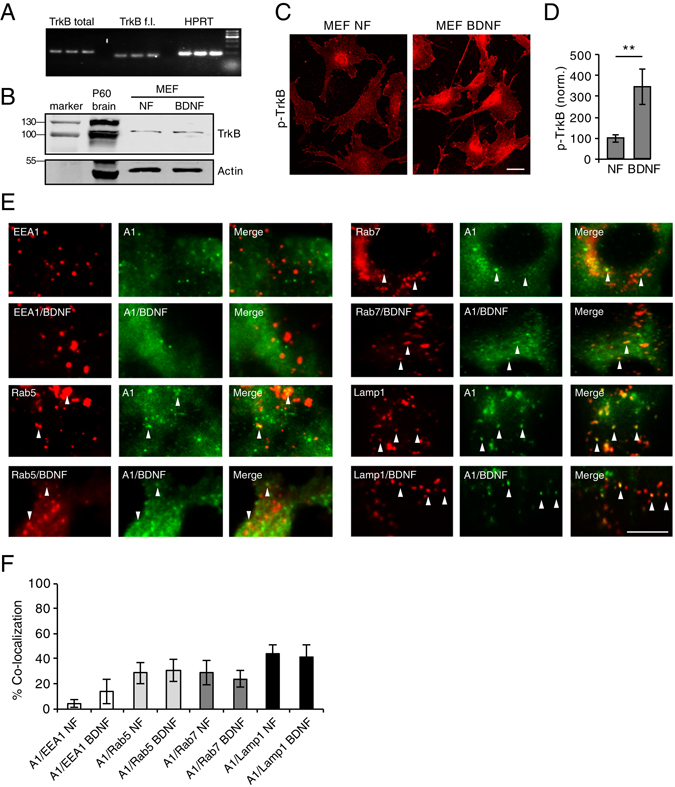
EndophilinA1 colocalizes with endosomes. (**A**) qPCR products of total TrkB (including full length and truncated isoforms), full length TrkB (TrkB f.l.) and HPRT as a control from mouse embronic fibroblast (MEF) lysates. (**B**) Western blot of TrkB in MEF lysates in control no factor (NF) conditions, and following treatment with BDNF. P60 brain lysate serves as a positive control, and actin as a loading control. (**C**) Images of pTrkB immunostaining in cultured MEFs in “no factor” (NF) untreated conditions, or following treatment with 100 ng/ml BDNF for 30 min; scale bar = 10 µm. (**D**) Quantitation of pTrkB signal in MEFs in NF and BDNF conditions; n-21 images per condition, error = SEM, significance determined by Student’s t-test. (**E**) Representative TIRF microscopy images of MEFs co-transfected with GFP-tagged EndophilinA1 and the RFP-tagged endosomal markers EEA1, Rab5, Rab7 or Lamp1, in the presence and absence of 100 ng/ml BDNF. Arrows indicate colocalization of EndophilinA1 (green) with endosomal markers (red). (**F**) Quantitation of colocalization of transfected GFP-tagged EndophilinA1 with RFP-tagged EEA1, Rab5, Rab7, or Lamp1 in control conditions with no factors (NF) or following addition of BDNF; n = 8–10 images per condition from 3 independent cell cultures, significance determined by unpaired two-tailed Student’s t-test comparing non-stimulated to stimulated conditions, error = SEM, scale bar = 10 µm.

To examine the possible localization of EndophilinAs to endosomal compartments, we co-transfected mouse embryonic fibroblasts (MEFs) from TKO mice^23^, with recombinant plasmids expressing EndophilinA1, A2 or A3 - tagged with GFP, together with recombinant plasmids expressing the endosomal markers EEA1, Rab5, Rab7, or Lamp1 - tagged with RFP. EEA1, Rab5, Rab7, and Lamp1 represent the main compartments involved in endosomal sorting and signaling, where EEA1 marks early endosomes, Rab5 marks early endosomes in the early stages of the sorting pathway, Rab7 marks late endosomes, and Lamp1 marks late endosomes/lysosomes. Because the sorting pathway involves transitions between endosomal compartments, there is some overlap in markers, e.g. Rab5 is gradually replaced with Rab7 in the transition from early to late endosomes, and Lamp1 is present on lysosomes, but up to 81% of Lamp1-positive compartments also contain Rab7^24^ and can therefore mediate endosomal signaling from these endo-lysosomal compartments. We examined co-localization of EndophilinAs with these endosomal markers in control cells and in cells treated with BDNF to activate TrkB receptors. Because a previous publication reported an absence of TrkB in MEFs^25^, we first examined TrkB expression. qPCR results indicated the presence of TrkB in MEFs (Fig. 2A), with a mean Cp value of 36. Western blots of MEF lysates also showed that TrkB was present in these cells (although at lower levels than in brain lysates) (Fig. 2B). In addition, phosphorylated TrkB was increased in MEFs by treatment with 100 ng/ml BDNF for 30 minutes (Fig. 2C,D). This suggests that functional TrkB is present in MEFs.

Analysis of TIRF microscopy images of co-transfected MEFs revealed that different EndophilinAs co-localize with distinct endosomal markers and are differentially recruited to endosomes in response to BDNF treatment. EndophilinA1 showed the least co-localization with endosomal compartments. EndophilinA1 co-localized with EEA1 only 5% before BDNF addition and 15% after, with Rab5 approximately 28% in both the absence and presence of BDNF, and with Rab7 28% and 23% in the absence and presence of BDNF, respectively (Fig. 2H,I). EndophilinA1 also co-localized with Lamp1 approximately 45% in both the absence and presence of BDNF (Fig. 2H,I). These results show co-localization of EndophilinA1 with endosomal compartments from early to late endosomal compartments with no significant change in the presence and absence of exogenous BDNF.

**Figure 3:**
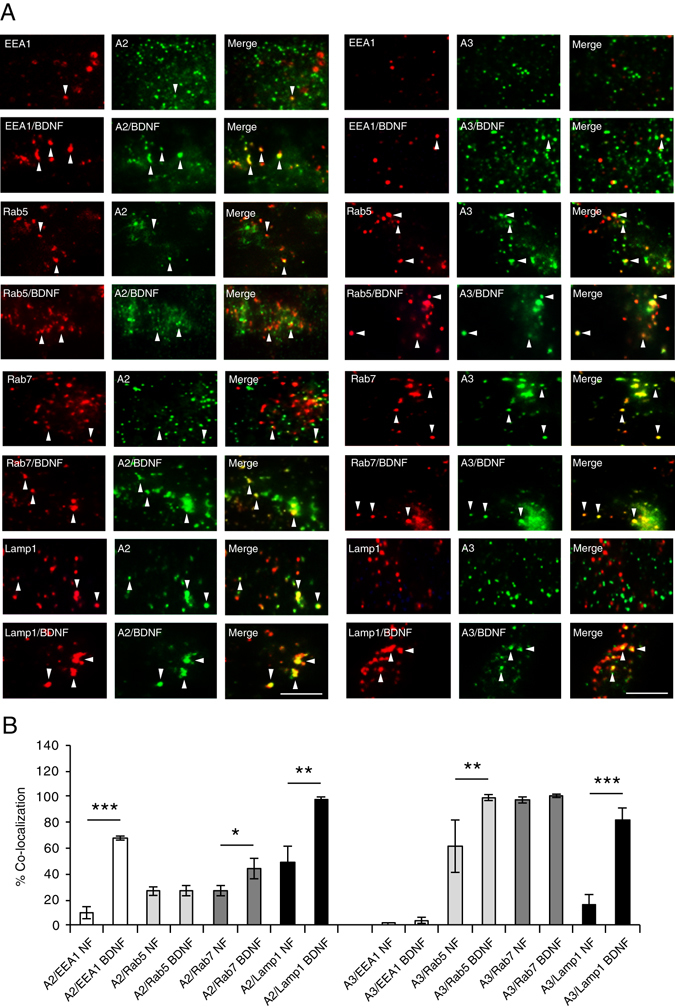
EndophilinA2 and A3 are recruited to endosomes in response to BDNF. (**A**) Representative TIRF microscopy images of MEFs co-transfected with GFP-tagged EndophilinA2 (left panels) or EndophilinA3 (right panels) and the RFP-tagged endosomal markers EEA1, Rab5, Rab7 or Lamp1, in the presence and absence of 100 ng/ml BDNF. Arrows indicate colocalization of EndophilinA (green) with endosomal markers (red). (**B**) Quantitation of colocalization of transfected GFP-tagged EndophilinA2, and A3 with RFP-tagged EEA1, Rab5, Rab7 and Lamp1; n = 8–10 images per condition from 3 independent cell cultures, significance determined by unpaired two-tailed Student’s t-test test comparing non-stimulated to stimulated conditions, error = SEM, *p < 0.05, **p < 0.01, ***p < 0.001, scale bar = 10 µm.

We then extended our analysis to the other two Endophilins, A2 and A3 and found that EndophilinA2 and EEA1 co-localized 10% before and 68% after stimulation with BDNF, while EndophilinA3 colocalized with EEA1 only 1% and 3% in the presence and absence of BDNF, respectively (Fig. 3A,B). Continuing with early endosomal marker Rab5; EndophilinA2 showed no change in co-localization with Rab5 in the absence (28%) or presence of BDNF (28%), while EndophilinA3 and Rab5 increased their co-localization from 61% in the absence of BDNF to almost 100% in the presence of BDNF. For late endosomes, EndophilinA2/Rab7 co-localized 47% and 67% in the absence and the presence of BDNF while EndophilinA3 co-localized approximately 98% with Rab7 both in the absence and presence of exogenously applied BDNF. EndophilinA2 increased co-localization with Lamp1 after application of BDNF from 60% to 98% and EndophilinA3 increased co-localization with Lamp1 from 16% in the absence of BDNF to 81% in the presence of BDNF. This demonstrates differential co-localization of EndophilinA2 and A3 to distinct endosomal compartments which changes in the presence of BDNF: EndophilinA2 is recruited to EEA1 and Lamp1-positive endosomes in response to BDNF, while EndophilinA3 is absent from EEA1 endosomes in both the presence and absence of BDNF, and is recruited to Rab5 and Lamp1-positive endosomes in the presence of BDNF. Both EndophilinA2 and EndophilinA3 co-localized with Rab7-positive endosomes at relatively high levels both with and without BDNF. In addition to being concentrated on endosomes in many cases, all EndophilinAs showed some degree of cytoplasmic localization, as previously reported^12, 13^.

**Figure 4:**
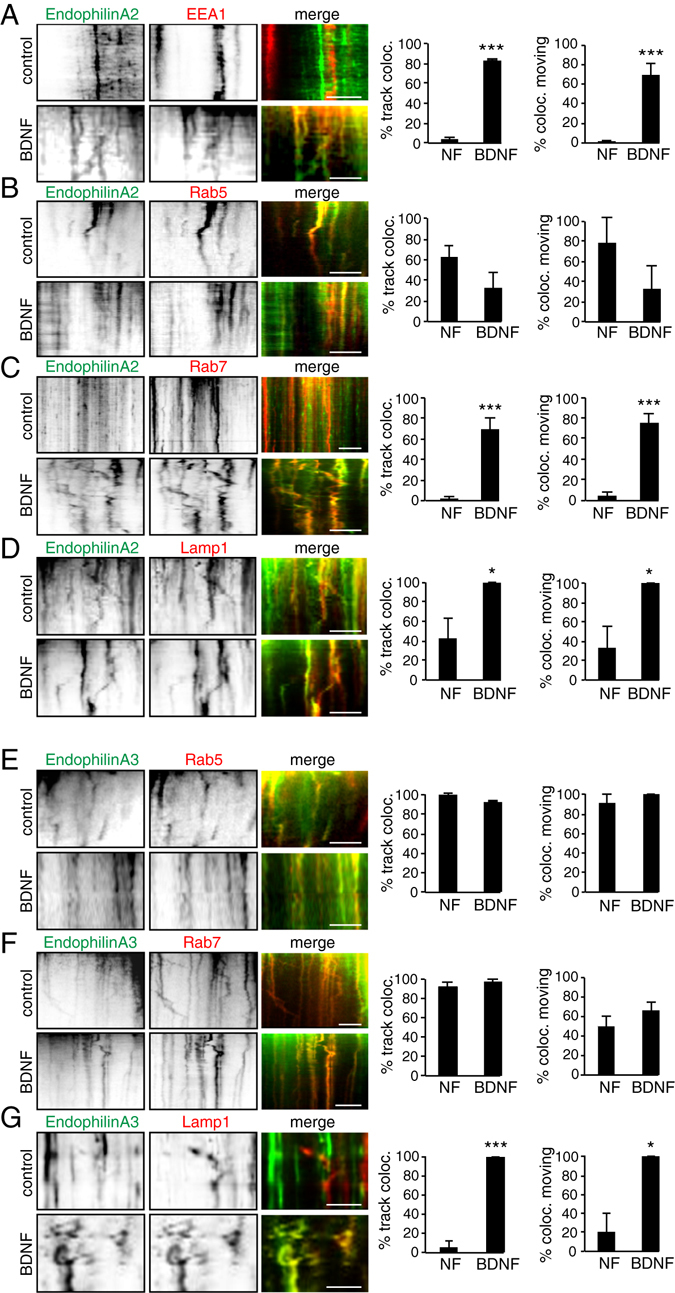
EndophilinA2 and EndophilinA3 co-traffic with endosomes. (**A**) Kymographs (left panels) of co-trafficking of EndophilinA2 and endosomal markers before and after application of 100 ng/ml BDNF in 5 minute TIRF time-lapse images of MEFs co-transfected with GFP-tagged EndophilinA2 and RFP-tagged EEA1, Rab5 (**B**), Rab7 (**C**), or Lamp1 (**D**). Quantitation of recruitment of EndophilinA2 to endosomes and mobility of EndophilinA2-positive endosomes (before or after administering 100 ng/ml BDNF) by percent of EndophilinA2 and endosomal kymograph tracks colocalizing, and percent of co-localizing mobile endosomes (A–D, right panels). (**E**) Kymographs of co-trafficking of EndophilinA3 and RFP-tagged Rab5 (**E**), Rab7 (**F**), or Lamp1 (**G**). Quantitation of recruitment of EndophilinA3 to endosomes before and after administering BDNF, by percent of tracks colocalizing, and percent of co-localizing mobile endosomes (E–G, right panels); n = 8–10 images per condition from 3 independent cell cultures, significance determined by unpaired two-tailed Student’s t-test non-stimulated to stimulated conditions, error = SEM, *p < 0.05, **p < 0.01, ***p < 0.001, scale bar = 10 µm.

In addition to co-localization, TIRF microscopy revealed co-trafficking of EndophilinAs with endosomes in conditions where co-localization appeared, and BDNF treatment increased the mobility of EndophilinA-positive endosomes. Since we found no significant increase in the localization of EndophilinA1 to endosomal compartments in the presence and absence of BDNF, we concluded a minor involvement of EndophilinA1 in potential endosomal sorting. We focused on EndophilinA2 and EndophilinA3-transfected MEFs where we found significant changes in colocalization after BDNF treatment. To investigate movement, we tracked EndophilinA and endosomal marker-positive puncta over time in kymographs, where vertical tracks represent stationary puncta and tracks that deviate from vertical represent movement over time. In the absence of BDNF, some EndophilinA2 signal was diffusely distributed in the cytosol (Fig. 4A). However, after stimulation with BDNF, EndophilinA2 signal overlapped with EEA1 positive tracks in kymographs, indicating recruitment of EndophilinA2 to EEA1-positive stationary, but also moving, endosomes (Fig. 4A). EndophilinA2 showed a trend toward decreased co-trafficking with Rab5 in the presence of BDNF (Fig. 4B). In the absence of BDNF, EndophilinA2 did not co-localize significantly with Rab7-positive late endosome tracks, but administration of BDNF recruited EndophilinA2 to Rab7-positive tracks (Fig. 4C, Suppl. Movies S1 and S2). In addition, co-trafficking of EndophilinA2 with Rab7-positive endosomes significantly increased in response to BDNF and the degree of co-trafficking was greater than EEA1-EndophilinA2 positive endosomes in the presence of BDNF. Additionally, we found that EndophilinA2 increased co-localization and co-trafficking with Lamp1 after application of exogenous BDNF (Fig. 4D, Suppl. Movies S3 and S4).

EndophilinA3 was highly co-localized with Rab5-positive compartments both before and after BDNF treatment (Fig. 4E). In contrast to EndophilinA2, we found over 91% co-localizing tracks of EndophilinA3 and Rab7 in kymographs in untreated conditions and over 97% co-localizing tracks in BDNF-treated conditions (Fig. 4F, Suppl. Movies S5 and S6). In addition, EndophilinA3 co-trafficked with Rab7-positive endosomes in both non-treated and BDNF-treated conditions (Suppl. Movies S5 and S6). Further, EndophilinA3, like EndophilinA2, increased co-localization and co-trafficking with Lamp1 after application of exogenous BDNF (Fig. 4G, Suppl. Movies S7 and S8).

These results reveal that all three EndophilinAs localize to and co-traffic with specific endosomal compartments in either the presence or absence of BDNF treatment, or both, indicating a potential role in activated TrkB receptor endosomal sorting.

### EndophilinAs tubulate late endosomes in the presence of BDNF

**Figure 5:**
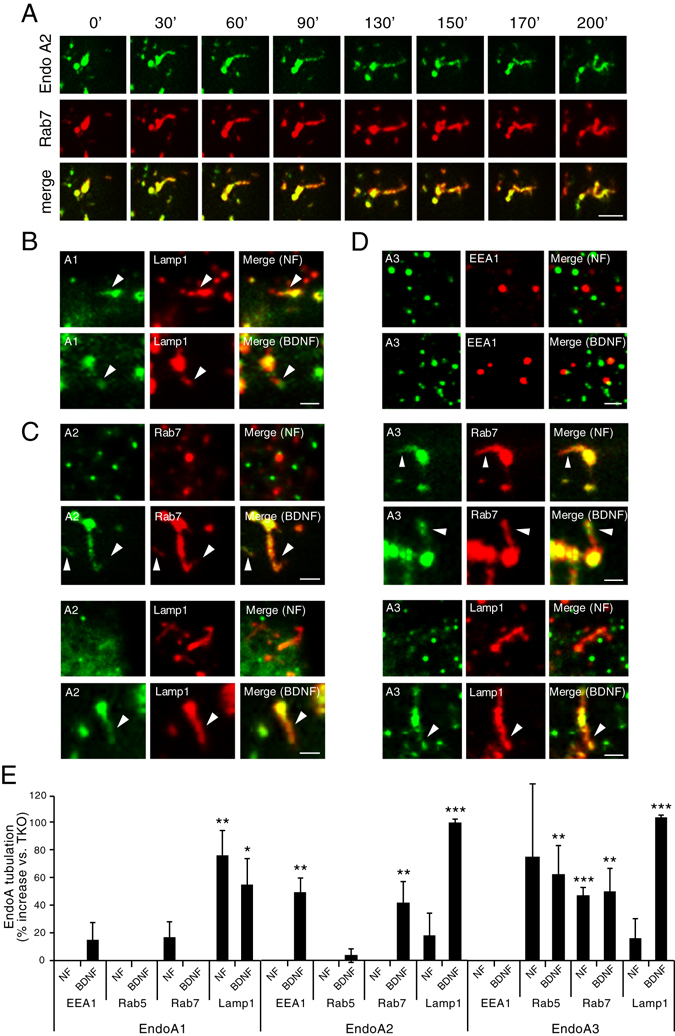
EndophilinAs tubulate endosomal compartments to which they are recruited. (**A**) Representative timecourse of a tubulation event of EndophilinA2 on a Rab7-positive endosome over 200 seconds in co-transfected EndophilinA TKO MEFs. Scale bar = 5 µm. (**B**) Example of tubulation events of EndophilinA1 on Lamp1-positive endosomes in the absence (NF) and presence of 100 ng/ml BDNF, of EndophilinA2 on Rab7 and Lamp1-positive endosomes in the absence (NF) and presence of BDNF (**C**), and of EndophilinA3 on EEA1, Rab7, and Lamp1-positive endosomes in the absence (NF) and presence of BDNF (**D**) Arrows indicate tubulation events in which endosomal markers and EndophilinA isoforms co-localize. Scale bars = 2 µm for (**B**–**D**). (**E**) Quantitation of the percent increase in tubulation of endosomal compartments by EndophilinA1, EndophilinA2, and EndophilinA3, compared to EndophilinA triple knockouts in control no factor (NF) conditions and following 100 ng/ml BDNF treatment; n = 8–10 images per condition from 3 independent cell cultures, significance determined by paired two-tailed Student’s t-test, error = SEM, *p < 0.05, **p < 0.01, ***p < 0.001, scale bar = 10 µm.

EndophilinA has the ability to bend and tubulate membranes to which it is bound, via its BAR domain^9^. Tubulation of endosomes marks the process of sorting by which receptors are routed in a C-terminal dependent manner through actin-stabilized microdomains, leading to eventual scission of vesicles from the tips of tubules^26^. Because EndophilinAs were localized to and recruited to endosomes, we tested if EndophilinAs are necessary to tubulate these endosomes. We used live TIRF microscopy of EndophilinA TKO MEFs co-transfected with RFP-tagged endosomal markers and GFP-tagged EndophilinAs, to allow a comparison of “knockout” endosomal tubulation events (RFP-labelled only) to tubulation induced by EndophilinAs (RFP and GFP co-labelled tubules, where a particular EndophilinA is present on endosomes). We found that specific EndophilinAs tubulate the specific endosomal compartments to which they are recruited. An example of a tubulation event (of EndophilinA2 and Rab7), is shown in Fig. 5A. We found that EndophilinA1 tubulates Lamp1-positive endosomes in the presence and absence of exogenous BDNF (Fig. 5B), while EndophilinA2 tubulates both Rab7 and Lamp1-positive endosomes in the presence of BDNF (Fig. 5C). In line with co-localization and co-trafficking data, EndophilinA3 did not tubulate EEA1-positive endosomes in the absence or presence of BDNF. But EndophilinA3 tubulated Rab7-positive endosomes in the presence and absence of BDNF, and tubulated Lamp1-positive endosomes only in the presence of BDNF (Fig. 5D).

To determine if tubulation of endosomes is specifically caused by EndophilinAs, we quantified the number of tubulation events of EndophilinA TKO endosomes (marked by RFP-tagged endosomal markers alone), compared to endosomes harboring EndophilinAs (Fig. 5E). We found that EndophilinAs specifically tubulate endosomes they co-localize with or are recruited to by BDNF. Tubulation was also seen in the absence of Endophilin, however, these events were restricted to early endosomes such as EEA1 and Rab5 where tubulation is likely mediated by the sorting nexins^15^. EndophilinA1 showed a trend towards increased tubulation of EEA1-positive endosomes, by 15% compared to TKO endosomes, in the presence of BDNF, and increased tubulation of Rab7-positive endosomes by 17% in the absence of BDNF. EndophilinA1 significantly increased tubulation of Lamp1-positive endosomes both in the absence (75%) and presence (55%) of BDNF (Fig. 5E). EndophilinA2 tubulated EEA1-positive endosomes, to a greater extent (50%) than EndophilinA1, and tubulated both Rab7 (42% increase compared to TKO endosomes) and Lamp1 (100% increase) -positive endosomes in response to BDNF, and showed a trend toward increased tubulation of Lamp1-positive endosomes to a lesser degree (20%) in the absence of BDNF (Fig. 5E). EndophilinA3 tubulated Rab5 and Rab7 endosomes (but not EEA1-positive endosomes) equally in both the presence and absence of BDNF (40–60%), and increased tubulation of Lamp1-positive endosomes (from 20% to 100%) following addition of BDNF (Fig. 5E).

### TrkB receptors accumulate within endosomes in the absence of EndophilinAs

**Figure 6:**
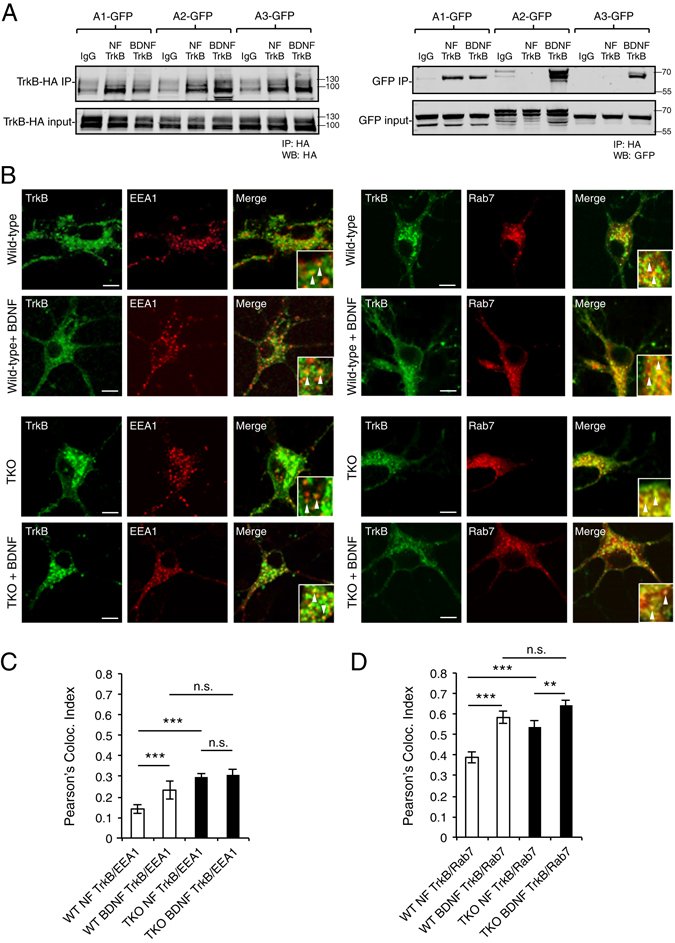
TrkB receptors accumulate in EEA1 and Rab7 positive endosomes in EndophilinA triple knockouts. (**A**) HA-conjugated ProteinA beads (or IgG control beads) were used to pull down HA-TrkB (left) and EndophilinA1, A2, or A3-GFP (right) in co-transfected Hek293 cells in the presence or absence of 100 ng/ml BDNF. (**B**) Representative images of wild-type and EndophilinA TKO mouse neuronal cultures transfected with TrkB-GFP and the RFP-tagged endosomal markers EEA1 or Rab7. Arrows indicate TrkB colocalizing with EEA1 or Rab7. Scale bar = 10 µm. (**C**) Quantitation of colocalization of TrkB-GFP with RFP-tagged EEA1 or Rab7 (**D**), in wild-type and EndophilinA TKO neurons. In EndophilinA triple knockout neurons, more TrkB was found in EEA1 and Rab7 positive endosomes in both the presence and absence of BDNF compared to control; n = 30 images from 4 independent cultures, significance determined by unpaired two-tailed Student’s t-test compared to non-stimulated control and between non stimulated TKO and BDNF treated TKO, error = SEM; **p < 0.01, ***p < 0.001.

The observation that EndophilinAs are recruited to and tubulate endosomal membranes in response to BDNF, led us to speculate that EndophilinAs may play a role in activated BDNF-TrkB receptor sorting. We first tested if EndophilinAs associate with TrkB by transfecting HEK293 cells with GFP-tagged EndophilinAs and HA-tagged TrkB and performing co-immunoprecipitation experiments in the absence and presence of BDNF. We found that HA-TrkB co-immunoprecipitated EndophilinA1, but not EndophilinA2 or EndophilinA3, in the absence of BDNF (Fig. 6A). However, in the presence of BDNF, HA-TrkB co-immunoprecipitated all three EndophilinAs; no binding was detected in IgG control beads that were not conjugated to the HA-antibody (Fig. 6A). This suggests that EndophilinAs bind either directly or indirectly to TrkB in the presence of BDNF, and further suggests that EndophilinA may modulate the sorting of activated BDNF-TrkB through endosomal compartments.

Activated TrkB traffics in Rab5 and Rab7-positive signaling endosomes targeted for long-range retrograde transport towards the soma to regulate survival signaling cascades^19, 27^. Our data showing that EndophilinAs bind to TrkB in the presence of BDNF, the ability of EndophilinA1, A2 and A3 to tubulate late endosomes, and the accumulation of Rab7-positive late endosomes in Endophilin TKO neurons led us to speculate that sorting and trafficking of activated TrkB receptors may be altered in EndophilinA TKO hippocampal neurons. To test this, we co-transfected hippocampal neurons in culture with GFP-TrkB and the endosomal markers RFP-EEA1 or RFP-Rab7 and examined their co-localization. We used overexpression in order to bypass the alterations in endogenous endosomal marker expression levels that occur in EndophilinA knockouts (Fig. 1C), and to reveal changes in receptor sorting between compartments. In control neurons, only about 13% of GFP-TrkB co-localized with RFP-EEA1 labelled early endosomes, into which receptors are sorted following ligand-induced endocytosis^28^ (Fig. 6C,D). Following stimulation with BDNF, GFP-TrkB and RFP-EEA1 co-localization was significantly enhanced (to about 23%) as expected, suggesting that in the there is a constitutive, low amount of internalization of receptors in response to endogenously released BDNF, while in the presence of exogenous BDNF, activated receptors are endocytosed in higher amounts and sorted into EEA1-positive endosomes. In contrast, EndophilinA TKO neurons showed higher co-localization of GFP-TrkB with RFP-EEA1 positive endosomes in control conditions than wild-type neurons (at levels similar to wild-type neurons treated with BDNF) and this co-localization was not further enhanced by BDNF, indicating stagnating or delayed trafficking of activated TrkB (Fig. 6C,D). From early endosomes, TrkB has been shown to transit into Rab5-positive early endosomes, and ultimately be sorted into Rab7-positive late endosomes, targeted for long-range trafficking to the soma^19, 27^. In accordance with this, we also found that GFP-TrkB co-localizes with RFP-Rab7 positive endosomes, in lower amounts (approximately 39%) in the absence of ligand and in significantly higher amounts (approximately 57%) after receptor activation following application of exogenous BDNF (Fig. 6C,E). We observed significantly higher co-localization between GFP-TrkB and RFP-Rab7 in EndophilinA TKO neurons than in wild-type neurons in control conditions, similar to what we observed for early endosomes marked with EEA1. However, co-localization of GFP-TrkB and RFP-Rab7 increased further in TKO neurons following addition of BDNF (Fig. 6D,E).

The high amounts of TrkB in EEA1 and Rab7 positive endosomes in EndophilinA TKO neurons - even in conditions without stimulation by exogenous BDNF - suggests that TrkB receptors previously activated by endogenous BDNF accumulate in early and late endosomes in EndophilinA TKO neurons. This further indicates that the endosomal sorting machinery is compromised due to the lack of EndophilinAs.

### BDNF-TrkB dependent signaling cascades are perturbed in EndophilinA knockouts

**Figure 7:**
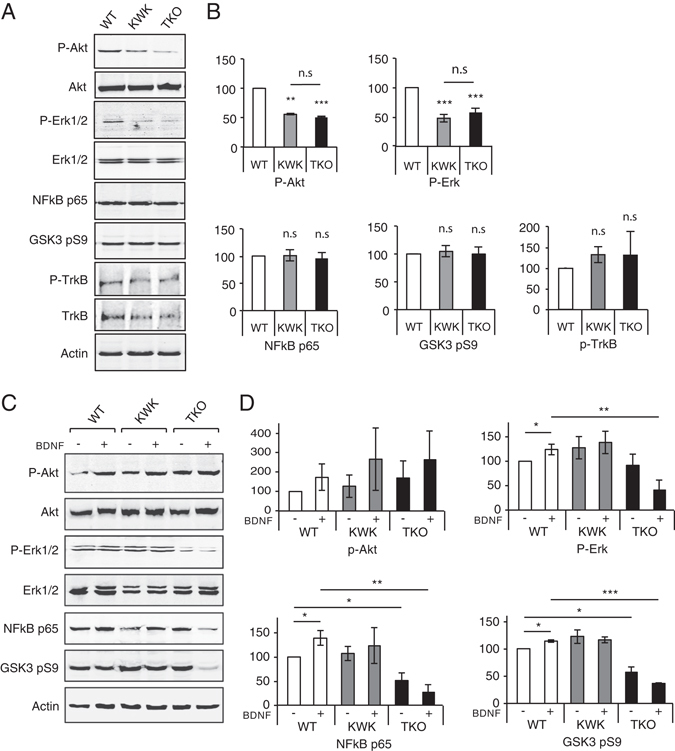
EndophilinA triple knockouts show changes in BDNF-mediated signaling. (**A**) Western blots of the indicated proteins in EndophilinA1/3 double knockout (KWK refers to Knockout, Wild-type, Knockout, for EndophilinA1, A2, A3), and EndophilinA triple knockout (TKO) brain lysates compared to wild-type. (**B**) Quantitation of P-Akt, P-Erk, NFkB p65, GSK3 pS9, and pTrkB protein levels in Western blots normalized to actin; n = 3 independent experiments, significance determined by unpaired two-tailed Student’s t-test comparing each condition to control, error = SEM, **p < 0.01, ***p < 0.001. (**C**) Western blots of the indicated proteins in EndophilinA1/3 double knockout (KWK refers to Knockout, Wild-type, Knockout, for EndophilinA1, A2, A3), and EndophilinA triple knockout (TKO) hippocampal cultures compared to wild-type in control and BDNF-treated conditions (100 ng/ml BDNF for 30 minutes). (**D**) Quantitation of P-Akt, P-Erk, NFkB p65 and GSK3 pS9 protein levels in Western blots normalized to actin; n = 3 independent experiments, significance determined by unpaired two-tailed Student’s t-test comparing each condition to control, error = SEM, **p < 0.01, ***p < 0.001.

Activated receptor sorting and trafficking is tightly linked to signaling. In a scenario where activated TrkB is sorted and trafficked incorrectly due to the absence of EndophilinAs, one would expect changes in signaling cascades regulated by BDNF-TrkB. To test this, we analysed Western blots of EndophilinA TKO brain lysates for levels of proteins that have been shown to be induced by BDNF-TrkB signaling to modulate neuronal survival, including p-Akt, p-GSK3 Ser9, p-ERK1/2 and NFkB p65. We found that p-Akt levels were reduced in EndophilinA TKO brain lysates (and in EndophilinA1/A3 double knockouts) compared to wild-type, consistent with a reduction in BDNF-TrkB survival signaling (Fig. 7A,B). Akt is activated by PI3 Kinase^1, 21^ downstream of BDNF-TrkB, and promotes survival^20^. P-Akt also activates the cAMP-responsive element binding protein (CREB) and nuclear factor-κB (NFκB), which are transcriptional regulators that may promote neuronal survival^22^. The levels of NFκB, and of p-GSK3 Ser9, which is inactivated by the PI3K/Akt pathway to promote survival signaling^29^ were unchanged in TKO brain lysates (Fig. 7A,B). However, in addition to reduced p-Akt levels, we also found reduced levels of p-ERK1/2 in EndophilinA TKO brain lysates, and in EndophilinA1/A3 double knockouts, compared to wild-type brain lysates (Fig. 7A,B). ERK activation has also been shown to promote neuronal survival downstream of BDNF-TrkB signaling^30^. We found no significant change in p-TrkB or total levels of TrkB in EndophilinA knockouts compared to wild-type brain lysates (Fig. 7A,B), suggesting that EndophilinA does not affect surface expression of TrkB receptors or extent of TrkB phosphorylation. Thus EndophilinA is likely important for BDNF-TrkB endosomal sorting, where key survival signaling cascades downstream of BDNF-TrkB are compromised in EndophilinA TKO brains.

Akt and Erk1/2 can be activated by several other growth factors and via alternate pathways, in addition to BDNF. To test if BDNF-TrkB survival signaling specifically, is compromised in EndophilinA knockouts, we analyzed the levels of BDNF-TrkB-induced survival proteins in EndophilinA TKO and wild-type neuronal cultures with and without BDNF treatment. We found that p-Akt levels were unchanged in EndophilinA TKO neurons (and in EndophilinA1/A3 double knockouts) compared to wild-type (Fig. 7C,D). This may be due to cultures having high activity, which increases the levels of p-Akt^31^, and could potentially mask the deficit in p-Akt we see in brain lysates from Endophilin TKOs (Fig. 7A,B). We further found that phospho-ERK was increased by BDNF treatment in wild-type cultures, but not in EndophilinA TKO cultures (Fig. 7C,D), indicating specific BDNF-TrkB dependent signaling deficits in EndophilinA TKOs. Similarly, NFkB p65 and GSK3 pS9 were both increased by BDNF treatment in wild-type cultures, but were present at lower levels in untreated EndophilinA TKO cultures and were not increased by BDNF (Fig. 7C,D), further suggesting BDNF-TrkB dependent survival deficits in EndophilinA knockouts. Interestingly, deficits in NFkB p65 and GSK3 pS9 were not observed in brain lysates, suggesting potential compensatory effects during development in brain.

### Neurons lacking EndophilinAs show reduced survival, which cannot be rescued by BDNF

**Figure 8:**
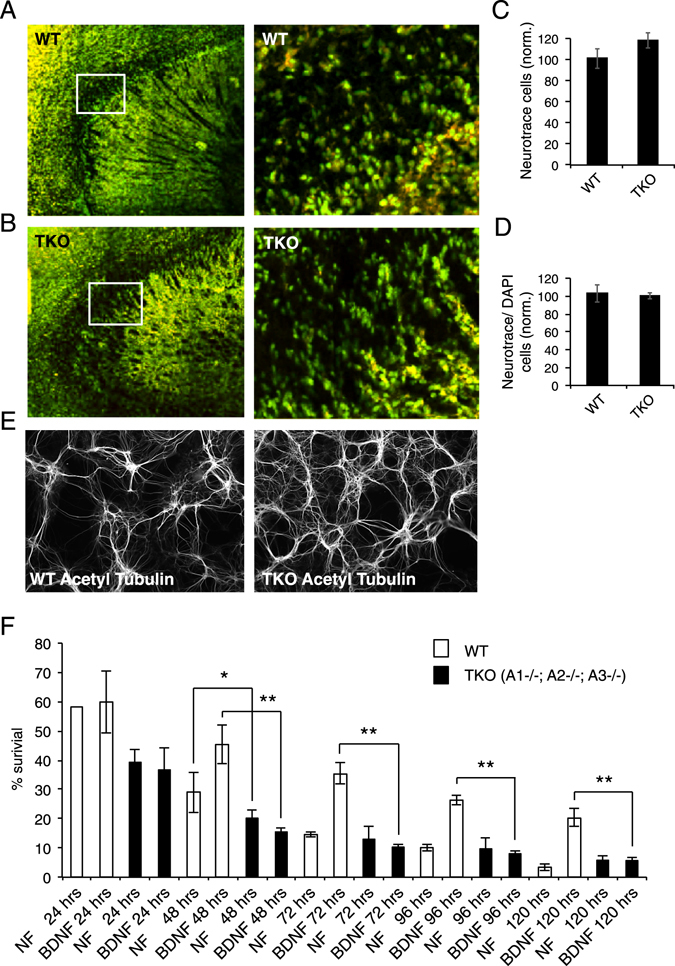
Survival is altered in low-density cultures of EndophilinA triple knockout neurons. (**A**,**B**) Brain sections of wild-type and EndophilinA triple knockout mice immunostained with Neurotrace (green) and DAPI (red) showed no obvious defects in overall structure of the hippocampus or the number of Neurotrace/DAPI-positive cells (quantified in **C**,**D**). (**E**) High-density neuronal cultures of wild-type and EndophilinA triple knockout neurons immunostained for acetyl tubulin. Both wild-type and EndophilinA TKO neurons formed networks and survived up to 14 days in culture. (**F**) Survival assay of wild-type and Endophilin A1-/-; A2-/-; A3-/- low-density cultures: EndophilinA TKO neurons showed higher rates of cell death compared to wild-type neurons after 48 hours in culture in the absence of BDNF. This increase in cell death in Endophilin A1-/-; A2-/-; A3-/- neurons could not be rescued by administration of 100 ng/ml exogenous BDNF compared to control during a 120-hour time course; n = 4 independent experiments, significance determined by unpaired two-tailed Student’s t-test, comparing each condition to numbers of initially plated neurons. Error = SEM, *p < 0.05, **p < 0.01.

Based on our finding that BDNF-TrkB endosomal sorting, trafficking and survival signaling cascades are perturbed in EndophilinA TKO neurons, we suspected that EndophilinA TKO neurons would have survival deficits. We first examined the overall architecture and number of neurons in the hippocampus of control and EndophilinA TKO mice using Neurotrace staining (Fig. 8A,B). We did not find any gross anatomical changes in the overall structure of the hippocampus, nor did we find a decreased number of Neurotrace-positive cells in TKO brain slices when normalized to DAPI staining per area (Fig. 8C,D). However, Murdoch *et al*.^32^ recently reported that EndophilinA TKO mice show higher Caspase activity in hippocampal neurons without gross anatomical changes- suggesting compensatory mechanisms. We also investigated neuronal survival in densely plated hippocampal neuronal cultures with approximately 50,000–75,000 cells per cm^2^, where neurons form synaptic connections. Acetylated tubulin staining to mark neuronal processes revealed no apparent difference in neuron number or morphology between wild-type and EndophilinA TKO cultures (Fig. 8E).

To uncouple the effects of synaptic connections and BDNF signaling, we next used an established BDNF-TrkB survival assay^33–36^, in which hippocampal neurons from EndophilinA TKO or wild-type mice were sparsely plated in culture. By plating low numbers of neurons, any released growth factors, which could act on neurons in a paracrine manner, are diluted in the media, and neurons are too far away from each other to make synaptic connections. Therefore, neurons are uncoupled from survival mediated through firing as well as potential neurotrophic factors. Neurons were counted 3 hours after plating to determine initial numbers and then every 24 hours, during which time cells begin to die due to a lack of concentrated growth factors. Our analysis revealed that neurons from EndophilinA TKO mice died more quickly compared to wild-type (Fig. 8F). Application of exogenous BDNF in this assay could rescue cell survival in wild type neurons but not in neurons lacking all three EndophilinAs. In control hippocampal neurons we observed increased survival in response to exogenous BDNF after 48–72 hours until 120 hours in culture, as expected. In contrast, EndophilinA TKO neurons did not respond to applied BDNF, showing a survival rate of only 15% after 48 hours compared to 45% in wild-type neurons. By 120 hours in culture, the survival rate of wild-type neurons was approximately 20% while only 6% of EndophilinA TKO neurons survived (Fig. 8F). Although a negative result, i.e. the lack of BDNF-induced survival of EndophilinA TKO hippocampal neurons, must be interpreted cautiously, this finding is consistent with a defect in BDNF-TrkB dependent survival signaling.

Together, our results indicate that EndophilinA TKO neurons have reduced activated TrkB receptor sorting through endosomal compartments and subsequent signaling. As a consequence, EndophilinA TKO neurons - when uncoupled from other survival mediating factors such as neuronal activity or high amounts of growth factor release - have decreased BDNF-TrkB dependent survival.

## Discussion

Our study uncovers a novel role of EndophilinAs in regulating the sorting of activated receptors through endosomal compartments. Using BDNF-TrkB receptor activation we found that specific EndophilinAs are recruited to distinct endosomal compartments in response to BDNF. EndophilinAs not only increase colocalization with endosomal compartments in response to BDNF, but also co-traffic with endosomal compartments and are necessary for BDNF-induced tubulation of the endosomal compartments to which they are recruited. In addition, activated TrkB receptors accumulate in endosomal compartments in EndophilinA TKO neurons, and Rab7-positive late endosomes accumulate in EndophilinA TKOs. Furthermore, BDNF-TrkB dependent survival signaling cascades are impaired in EndophilinA TKOs, and EndophilinA TKO neurons have reduced survival in a BDNF-dependent survival assay, compared to wild-type neurons. Interestingly, EndophilinA TKO neurons also had less Map2 in cell bodies and more punctate Map2 signal in neuronal processes compared to WT controls. TrkB-mediated BDNF signaling promotes the expression of MAP2 and microtubule assembly^37–40^. Thus it is possible that Endophilin KO neurons have decreased MAP2 levels due to deficiencies in BDNF signaling.

To date, EndophilinA has only been reported to play a role in endocytosis - of synaptic vesicles or receptors - from the plasma membrane^5–7^. Our data indicate that EndophilinA is important for endosomal sorting of activated TrkB receptors, and not for their endocytosis. An endocytic defect in EndophilinA TKOs would result in less activated TrkB receptors in early endosomal compartments of EndophilinA TKO neurons; We found the opposite – an increase in activated TrkB receptors in early endosomal compartments – indicating impaired endosomal sorting of activated TrkB, and not impaired endocytosis. A previous study found that EndophilinA1 binds retrolinkin, which is necessary for BDNF-TrkB endocytosis^41^. It was proposed that retrolinkin and EndophilinA1 act together to promote endocytosis of activated receptors. The authors found that EndophilinA1 knockdown did indeed impair BDNF-dependent ERK signaling and dendrite outgrowth. However, EndophilinA1 was not required for endocytosis of BDNF-TrkB or localization of activated Trk receptors to early APPL1 positive endosomes. These results are consistent with our finding that EndophilinAs promote late endosomal sorting, and not BDNF-TrkB endocytosis.

Interestingly, EndophilinB1, which has approximately 30% homology to EndophilinA1, has been implicated in NGF-TrkA trafficking. Unlike EndophilinA1, EndophilinB1 was reported to colocalize predominantly with early EEA1-positive endosomes and not with late Rab7 or Lamp1-positive endosomes. Knockdown of EndophilinB1 caused enlargement of early EEA1-positive vesicles in PC12 cells - but only after NGF treatment, which the authors hypothesized was due to increased endocytosis or reduced recycling back to the surface, rather than deficiencies in endosomal sorting. Nonetheless, similar to our findings regarding EndophilinA and BDNF-TrkB, this study found that knockdown of EndophilinB1 reduced ERK signaling and inhibited NGF-induced neurite outgrowth in PC12 cells^42^. EndophilinB1 may, however, have additional effects, given that loss of EndophilinB1 reduces autophagosome formation and was actually found to prolong cell survival under starvation conditions in another study^43^. In another recent study, neurite extension in response to NGF was inhibited in EndophilinA triple knockdown PC12 cells^7^. However this was also attributed to defective endocytosis and not to defective endosomal sorting, which was not examined in this study.

EndophilinA knockout mice accumulate clathrin-coated vesicles^6^; hence, one would expect these vesicles to be unable to fuse with endosomes. In this context, endosomal numbers should remain the same or decrease in EndophilinA knockout mice compared to controls. Our unexpected observation that EndophilinA TKO mice have increased numbers of Rab7-positive endosomal structures raised the possibility of specific roles of EndophilinAs in endosomal sorting. Cells lacking the WASH complex, a part of the endosomal sorting machinery^15^, sustain a collapse of the endo-lysosomal system leading to enlarged endosome-like compartments in which cargo accumulates^44, 45^, mimicking the effect of EndophilinA triple knockouts. We found that each EndophilinA shows a different degree of localization with endosomal compartments in non-stimulated and BDNF-stimulated conditions. EndophilinA1, reported to be the most abundant in the brain^3, 6, 46^ showed the least localization with endosomes, in the presence or absence of BDNF. However, EndophilinA2 was recruited to EEA1, Rab7 and Lamp1-positive endosomes in the presence of exogenous applied BDNF. EndophilinA3 was recruited to Rab5 and Lamp1-positive endosomes in the presence of BDNF, and co-localizes and traffics with Rab7 in the absence and presence of BDNF. Importantly, EndophilinAs were necessary for BDNF-induced tubulation of endosomal compartments to which they were recruited.

Because the sorting pathway involves transitions between endosomal compartments, there is some overlap in markers, i.e. the majority of Lamp1-positive compartments have also been reported to contain Rab7^24^ and could therefore represent endo-lysosomal compartments capable of signaling. In this case, the recruitment of EndophilinAs to Lamp1-positive compartments may be important for endo-lysomal signaling. Alternatively, the Lamp1-positive compartments to which EndophilinA is recruited could represent mature lysosomes, where EndophilinA is important for the degradation of activated TrkB receptors. The latter possibility seems less likely, however, since we found that EndophilinA knockout neurons have BDNF-TrkB-dependent deficits in survival.

EndophilinA may be a novel component of the retromer complex, which sorts activated receptors through endosomal compartments^16^. After activated receptor cargo enters tubular microdomains of endosomal sorting platforms, vesicles are formed at the very tip of the tubule via scission by recruited dynamin. These vesicles harbor the endosomal markers Rab5 and Rab7^16^, which destines them for recycling, long-range trafficking, or degradation^15, 16^. The sorting nexins are believed to mediate retromer tubule and vesicle formation through their C-terminal Bin-Amphiphysin-Rvs (BAR) domains and intrinsic self-assembling activity. EndophilinAs contain an N-terminal BAR domain, which binds to and can tubulate membranes and a C-terminal SH3 domain, which interacts with dynamin and synaptojanin. Therefore EndophilinA is capable of both sensing and inducing membrane curvature^9^. Given its structural similarity to sorting nexins and our finding that it is localized to endosomes, EndophilinA may represent a new member of the retromer complex.

Accumulation of TrkB in both EEA1 and Rab7-positive endosomes could arise from two potential problems: 1) constitutively endocytosed TrKB activated by endogenous BDNF is not sorted from early to late endosomes and as a consequence TrkB accumulates within early EEA1- positive endosomes. This would explain why the amount of activated TrkB within EEA1 positive endosomes was not further increased after adding exogenous BDNF. Or, 2) sorting of endocytosed TrkB still happens but is slowed down in the absence of EndophilinAs. This would explain why TrkB is present at higher levels in Rab7-positive late endosomes in knockouts and why administering BDNF further increases TrkB in Rab7 endosomes in EndophilinA knockouts. In this case, sorting may still happen - possibly due to a compensatory role of Snexins - but is slowed down in the absence of EndophilinAs.

BDNF-TrkB activates PI3 kinase, which activates Akt^1, 21^ to promote survival^30, 47, 48^. A decrease in p-Akt in EndophilinA TKO brain lysates is therefore consistent with a reduction in BDNF-TrkB mediated survival signaling in EndophilinA TKOs. P-Akt levels were unchanged in cultured EndophilinA TKO neurons compared to wild-type, however, possibly because p-Akt levels are increased by neuronal activity^31^, which may be higher in cultures than in brain and could potentially mask the deficit in p-Akt seen in brain lysates from Endophilin TKOs. ERK activation, which is mediated by late endosomes^49^ was compromised in EndophilinA TKOs. ERK activation promotes neuronal survival downstream of BDNF-TrkB signaling: BDNF-TrkB activates the GTPases Ras and Rap1, which activate B-Raf to cause sustained activation of the MEK/MAPK signaling cascade^50–52^ to phosphorylate and activate ERK^30^. The decrease in p-ERK we found in EndophilinA TKOs is therefore consistent with the decreased survival of EndophilinA TKO neurons. Phospho-ERK was increased by BDNF treatment in wild-type cultures, but not in EndophilinA TKO cultures, indicating specific BDNF-TrkB dependent signaling deficits in EndophilinA TKOs. NFκB, a transcriptional regulator that can promote neuronal survival^22^, and p-GSK3 beta Ser9, which is inactivated by the PI3K/Akt pathway to promote survival signaling^29^, were both increased by BDNF treatment in wild-type cultures, but were present at lower levels in untreated EndophilinA TKO cultures and were not increased by BDNF, further indicating BDNF-TrkB dependent survival deficits in EndophilinA knockouts. Interestingly, deficits in NFκB p65 and GSK3 pS9 were not observed in brain lysates, suggesting potential compensatory effects during development in brain.

We found - using an established BDNF-TrkB survival assay to uncouple BDNF signaling from other survival-promoting mechanisms^33–36^ - that EndophilinA TKO neurons died more quickly compared to wild-type neurons. Moreover, while application of exogenous BDNF rescued cell survival in wild-type neurons, BDNF failed to rescue survival in EndophilinA TKO neurons. In addition to our finding, it was recently reported that Endophilin TKO neurons have increased caspase activity^32^. This suggests that the lack of EndophilinA in hippocampal neurons increases their susceptibility to neuronal death, likely by impairing receptor sorting and subsequent survival signaling.

EndophilinA TKO mice die shortly after birth and their brain (and overall body size) is smaller than wild-type mice^6^. However, our analysis of brain slices of wild-type and EndophilinA TKO mice showed no gross defects in cell numbers or overall architecture of the hippocampus, and we did not find a decrease in number of neurons in densely plated cultures from EndophilinA knockouts. This finding was in line with the recent paper of Murdoch *et al*.^32^, where the authors find increased caspase activity in TKO brain slices without gross changes in the overall anatomy, suggesting that either TKO mice die before neuronal death is visible or that compensatory mechanisms are present. The latter is in agreement with the finding that brains of BDNF knockout mice- where BDNF was excised in postmitotic neurons under a tau driver- show no gross changes in the overall structure, volume and size of the hippocampus, suggesting that BDNF is not essential for postnatal neuronal survival in the brain^53^. Synaptic connections and neuronal activity could compensate for the loss of EndophilinAs and reduced BDNF trafficking and signaling in brain slices and high-density cultures, since synaptic transmission in cultured EndophilinA TKO neurons was decreased but not abolished^6^. However, mutations in EndophilinAs may have additional effects in neurodegenerative or disease states in the brain. It has been reported in neuropsychiatric diseases that mutations in a single gene often have only a small contribution to the disease risk, while the combinations of risk polymorphisms have a cumulative effect^54^.

## Materials and Methods

All research involving animals was approved by and done in accordance with the Institutional Animal Care and Ethics Committees of Goettingen University (T10.31) and with German animal welfare laws, and in accordance with the Animals Scientific Procedures Act of 1986 (UK). EndophilinA -/- and control C57BL/6 mice were used at P0 from both sexes for neuronal cultures as well as for MEF-preparation.

### Genotyping of EndophilinA knockouts

EndophilinA1 genotyping was performed using the primers EndoA1-forward: CCACGAACGAACGACTCCCAC, EndoA1-reverse-WT: CGCACCTGCACGCGCCCTACC, and EndoA1-reverse-KO: TCATAGCCGAATAGCCTCTCC, with PCR cycling of 95 °C 5 min; 95 °C 30 s, 59.5 °C 40 s (x35); 72 °C 1 min; 72 °C 10 min; 10 °C ∞, yielding bands of 384 bp for WT and 950 bp for EndophilinA1 KO. EndophilinA2 genotyping was performed using the primers EndoA2-forward-WT: CTTCTTGCCTTGCTGCCTTCCTTA and EndoA2-reverse-WT: GCCCCACAACCTTCTCGCTGAC, or EndoA2-forward-KO: CCTAGGGGCTTGGGTTGTGATGAG, EndoA2-reverse-KO: CGTATGCAGCCGCCGCATTGCATC, with PCR cycling of 95 °C 5 min; 95 °C 30 s, 60 °C 45 s (x37); 72 °C 90 s; 72 °C 7 min,; 10 °C ∞, yielding bands of 1280 bp for WT and 1000 bp for EndophilinA2 KO. EndophilinA3 genotyping was performed using the primers EndoA3-forward: CTCCCCATGGTGGAAAGGTCCATTC, EndoA3-reverse-WT: TGTGACAGTGGTGACCACAG, and EndoA3-reverse-KO: CAACGGACAGACGAGAGATTC, with PCR cyclng of 95 °C 5 min; 95 °C for 30 s, 58 °C for 30 s, 72 °C 50 s (x35); 72 °C 10 min; 10 °C ∞, yielding bands of 325 bp for WT and 465 bp for EndophilinA3 KO. Examples of genotyping results are shown in Suppl. Fig. 2.

### RNA Isolation and RT-PCR

Mouse embryonic fibroblasts were homogenized with 1 ml TRI reagent (Sigma Aldrich), incubated at room temperature for 5 min, and 0.2 ml of chloroform was added. Samples were shaken vigorously for 15 seconds, and allowed to settle for 2–15 min at room temperature. Samples were then centrifuged at 12,000 g for 15 min at 2–8 °C. The upper aqueous supernatant was transferred to a new tube, an equal volume of isopropanol added and mixed by gentle inversion followed by incubation at −20 °C for 1 h. Samples were then centrifuged at 13,000 g for 30 min at 2–8 °C. The supernatant was discarded and the RNA pellet washed with 1 ml ice cold 70% ethanol. After washing, a final centrifugation step of 5 min at 4 °C and 12,000 g was performed, supernatant was discarded and the RNA pellet air-dried and dissolved in DNase- and RNase-free water. Samples were treated with DNase I (Ambion) to eliminate any remaining chromosomal DNA. Purity and concentration of RNA was determined by a spectrophotometer (NanoDrop ND-1000) with ND-1000 v3.5.2 software (Peqlab Biotechnologie). Only isolated RNA with an integrity value of more than 8 was used. cDNA was synthesized using 5–7 µg of the isolated RNA: RNA samples were diluted with DNase- and RNase-free water to a final volume of 11 µl and mixed with 9 µl of the cDNA synthesis reaction mix (4 µl 5x reaction buffer, 0.5 µl RNase inhibitor, 2 µl deoxynucleotides, 0.5 µl reverse transcriptase, 2 µl random hexamer mix from Roche Applied Science) for a final volume of 20 µl. RNA was reversed transcribed with the Transcriptor High Fidelity cDNA synthesis kit (Roche Applied Science) under the following conditions: 1 × 10 min. 25 °C, 1 × 30 min. 55 °C, 1 × 5 min. 80 °C, followed by a hold at 4 °C. A reverse transcription - polymerase chain reaction (RT-PCR) without reverse transcriptase served as a negative control. Quantitative PCR (q-PCR) was performed on a light cycler 480 system (Roche Applied Science) using LC480 SYBR green Master mix (Roche Applied Science). Forward and reverse primers, respectively, were used for identification of total TrkB; including full length and truncated forms (ttctgcctgctggtgatgt, tccagtgggatcttatgaaaca), and for full length TrkB specifically (tgcccagagcaggataagat, aaagtccttgcgtgcattgt) All primers were synthesized by and purchased from Sigma-Aldrich.

### Culture of Hippocampal Neurons

Hippocampi were removed and transferred to ice-cold HBSS buffer and kept on ice during dissection. Hippocampi were then transferred into Papain-enzyme solution and incubated for 30 min at 37 °C.

During the incubation, 1.5 ml plating medium (Neurobasal containing B27, Pen/Strep and 10% FBS) was filled into each well of 12-well plates containing poly-L-lysine coated cover slips. After incubation, enzyme solution was removed and 5 ml plating medium added and incubated for 5 min at 37 °C for neutralization. Plating medium was removed and hippocampi were washed twice with plating medium or HBSS. 2 ml plating medium was added per brain and the tissue was disassociated with a 1 ml pipette. 400 μl of cell suspension was added to each well, thereby splitting two hippocampi into 5 wells. The following day, the plating medium was removed and 1.5 ml prewarmed (37 °C) neuronal medium (Neurobasal containing B27, Pen/Strep) was added to each well. If the culture contained a lot of dead cells, wells were washed with Neurobasal prior to addition of neuronal medium. Neurons were then kept at 37 °C, 5% CO2 and 100% humidity until use.

### Transfection with Lipofectamine 2000 reagent (Invitrogen)

RFP-tagged Rab5 and Rab7 were obtained from A. Helenius, Tag-RFP-T-EEA1 from S. Corvera, TrkB-GFP from R. Segal, Lamp1-RFP from W. Mothes and the HA-TrkB construct from Y.A. Barde all through Addgene. GFP-tagged endophilinA1, A2, A3 constructs were obtained from I. Milosevic/P. De Camilli^23^. Transfection was performed on DIV 4/5 hippocampal neurons, according to the manufacturer’s instructions. For solution A, 1 μl of Lipofectamine 2000 was added to 100 μl Opti-MEM medium (per well) and incubated for 5 min at RT. For solution B, 1 μg of the desired plasmid was added to 100 μl of Opti-MEM medium (per well). Then both solutions were mixed and incubated for 20 min at RT. In the meantime 2/3 of the neuronal medium from each well was removed and stored at 37 °C. After the incubation time, 200 μl of the transfection mixture was added to each well and incubated for 75 min at 37 °C. Finally, the transfection mixture was removed and the stored neuronal medium was added back to the cultures.

### Calcium phosphate transfection of HEK293 cells

HEK293 cells (used for IP of Endophilins and TrkB) were transfected at 80% density. For one 10 cm tissue culture dish, 10 µg each of TrkB-HA and EndoA-GFP were mixed with 133 µl 2.5 M CaCl_2_, in a total volume of 1.3 ml with dH_2_O. An equal volume of 1.3 ml of 2X HBS (280 mM NaCl, 1.5 mM Na2HPO4, 50 mM Hepes, pH 7.05) was then added dropwise while under gentle vortex. The mixture was then added dropwise to the HEK293 cells cultured in 10 ml of DMEM containing 10% FBS and Pen/Strep, and left in the media until cells were harvested 2–3 days after transfection.

### Lonza AMAXA transfections

Mouse embryonic fibroblasts from WT and Endophilin TKO were counted and 1.5 million cells were transferred into a 15 ml Falcon tube. After aspirating all media, 95 µl of the prepared AMAXA transfection solution was added to the cells together with 4 µg of the desired plasmids. Both were gently mixed and transferred into the transfection cuvette and transfection was performed with the respective program for mouse embryonic fibroblasts of the AMAXA electroporating machine. After transfection, cells were transferred into 1 ml of media (DMEM containing Pen/Strep and 10% FBS) and 50,000 cells were plated on poly-L-lysine coated cover slips in a 12 well plate.

### TIRF microscopy

Transfected MEF cells were trypsinized and re-plated on a MatTek 35 mm glass bottom poly-D-lysine coated tissue culture dish. On the day of imaging, MEF cells were stimulated for 30 minutes with either recombinant BDNF (R&D Systems) or with PBS containing 0.1% Albumin and placed on a Zeiss AxioObserver Z1 TIRF Microscope with an Evolve CCD camera (Photometrics) using the 100x objective. 5-minute time-lapse recordings (with pictures taken in 10 sec. intervals) were made from which we analyzed co-localization and generated Kymographs. Co-localization was evaluated using the ImageJ cell counter plugin to label red (endosomal markers), green (EndophilinA) and red/green (yellow) puncta, which were normalized to the total number of endosomal puncta. Kymographs were generated using Metamorph software (Molecular Devices). For analysis of kymographs, EndophilinA and endosomal markers that colocalized for at least 2.5 minutes continuously during each 5 minute timelapse, were considered a “colocalizing track”. To compare co-movement of EndophilinAs with different classes of endosomes, we quantified the percent of moving tracks, where moving defined as a deflection greater than 20 degrees from vertical for at least 30 seconds. Tubulation events were measured in time lapse videos as described above using the metamorph software (Molecular Devices): endosomes extending a tubular microdomain were circled and marked during the time of each time-lapse movie. Each event was analyzed in the red channel (Rab-GTPase), in the green channel (EndophilinA) and in merge of both. Number of events was normalized to endosomes tubulating only in the red channel, therefore in the absence of EndophilinAs (% of tubulation over TKO).

#### Antibodies

Antibodies used were: EEA1 (rabbit) # PA5-17228 from Thermo Scientific; Rab5 (mouse) # 108001 and Rab7 (rabbit) # 320003 from Synaptic Systems; Lamp1 (rabbit) #9091, Akt (rabbit) # 4685, p-Akt (rabbit) # 5012, p-NFkB p65 (rabbit) # 3033, GSK3b Ser9 (rabbit) #12456, tubulin (rabbit) #5666, acetylated tubulin (rabbit) #5335, ERK (MapK 42/44; rabbit) # 9102, p-ERK (p-MapK 42/44; rabbit) # 9101 from Cell Signaling; Map2 (chicken) # 5392 Abcam; TrkB (rabbit) # 07-225, actin (mouse) #MABT826 from Millipore; pTrkB (kindly provided by Moses Chao, New York University).

### Immunocytochemistry

Hippocampal neurons were fixed on DIV8 in 4% PFA and kept in PBS at 4 °C until use. Cells were blocked in 1% cold fish gelatine, 0.1% Triton and 2% BSA for at least 60 min on a shaker at RT. Cells were then incubated in primary antibody (1/500) overnight in blocking solution at 4 °C. After three washes of approximatively 5 minutes in PBS, secondary antibody was applied for 120 min at RT. Cells were washed for 10 min with DAPI in PBS (dilution: 1:5000), rinsed once with PBS followed by a quick rinse in ddH_2_O and then the coverslips were immediately mounted using Mowiol 4–88.

Brain slice cryosections from 4% PFA-fixed brains stored in sucrose at −20 °C were rehydrated with PBS for 40 min followed by washing for 5 minutes with PBS plus 0.1% Triton X-100. Sections were then incubated with NeuroTrace stain in PBS (1:200) for 20 min. Following this incubation, sections were washed again in PBS plus 0.1% Triton X-100, followed by a 2 hour wash in PBS at RT. Sections were then counter-stained with DAPI, washed two times for 5 minutes each and mounted in Mowiol 4–88.

### Survival Assay

Hippocampal neurons (WT and Endophilin TKOs) were dissected as described above, but plated into 35 mm tissue culture dishes coated with poly-L-lysine, at a density of approximately 2000 neurons per dish. After an initial count, which was performed 2 hours after plating for neurons to settle on the bottom of the culture dish, half of the dishes were treated with 100 ng/ml BDNF, and half of the dishes served as controls. The number of neurons within a pre-defined 12 × 12 mm area in the middle of the culture dish were counted 24 hours after plating and again at 48, 72, 96 and 120 hours. The number of neurons surviving at these times was expressed as a percentage of the initial number of neurons. Triplicate cultures were analyzed for all conditions and the data shown are compiled from three separate experiments.

### Brain homogenates and Western blots

Whole brain or cortex samples for Western blotting were prepared by homogenizing brain tissue stored at −80 °C. Shortly before use tissue was placed in −20° blocks until being quickly added to 2 ml Eppendorf tubes containing metallic beads and 1080 ul of homogenization buffer (1 protease inhibitor tablet (Roche) and 5 phospho-stop phosphatase inhibitor tablets (Roche) pulverized and dissolved in 10 ml dH_2_O, to which 0.0079 g dithiothreitol, 3.75 ml 2 M NaCl, 100 µl 0.5 M EDTA, and 1 ml 1 M HEPES at pH 7.4 were added, and the total volume brought to 50 ml with dH_2_O) immediately before tissue disruption on the bead mill (Retsch MM400). 120 µl 20% sodium dodecyl sulfate (SDS) was then added to completely solvate lipids and cholesterol, with a final concentration of 2% SDS in a final total volume of 1200 µl. The homogenate was then subjected to vigorous mixing by pulling it 15–30× through a 27-gauge needle into a 1 mL syringe to break up any semi-solid remnants and shear viscous DNA strands so that the mixture was fluid and homogeneous for use in SDS-PAGE gels.

Sample concentration was then quantified with a Pierce BCA kit to determine protein levels. Samples were prepared for SDS-PAGE by adding 2x sample buffer and boiling for 10 minute followed by loading on a 10%, 12% or 15% SDS-PAGE gel, depending on the target protein size.

### Immunoprecipitation

HEK293 cells (plated in 10 cm culture dishes in DMEM containing 10% FBS and penicillin/streptomycin) were co-transfected with TrkB-HA and EndophilinA-GFP constructs using the calcium phosphate transfection method. Two days after transfection cells were stimulated either with 100 ng/ml BDNF or 0.1% BSA (as a control) for 20 minutes. Following stimulation, cells were washed in ice-cold PBS. Cells were then lysed with 1 ml 10 mM Tris-HCl; pH = 7.5, 150 mM NaCl, 0.5 mM EDTA and 0.5% NP-40, centrifuged at 13,000 rpm for 15 minutes and the supernatant transferred into a new Eppendorf tube. 100 µl of supernatant was taken for the input control and mixed with 20 µl of 6X sample buffer. The remaining lysate was added to ProteinA magnetic beads pre-coated with sheep anti-rabbit IgG (Lifetech) that were then washed 3 times with PBS and incubated with HA-antibodies (or without HA antibodies for IgG control beads) overnight. Lysate and HA antibody-conjugated beads were incubated for 4 hours, the supernatant was removed, beads were washed 3 times with PBS, and 120 µl of 2X sample buffer was added to the beads. All samples were boiled for 10 min and then loaded on a 10% SDS-PAGE gel for Western blotting.

## Electronic supplementary material

Supplemental video 1

Supplemental video 2

Supplemental video 3

Supplemental video 4

Supplemental video 5

Supplemental video 6

Supplemental video 7

Supplemental video 8

Dataset 1
