# Supplementary material for: EndophilinAs regulate endosomal sorting of BDNF-TrkB to mediate survival signaling in hippocampal neurons
Source: Sci Rep. 2017 May 19;7:2149. doi: 10.1038/s41598-017-02202-4 (PMC5438371; doi:10.1038/s41598-017-02202-4)
Supplement: Supplementary file 9 — Dataset 1 [file 41598_2017_2202_MOESM9_ESM.docx]

**EndophilinAs regulate endosomal sorting of BDNF-TrkB to mediate survival signaling in hippocampal neurons**

Katja Burk, John D. Murdoch, Siona Freytag, Melanie Koenig, Vinita Bharat, Ronja Markworth, Susanne Burkhardt, Andre Fischer and Camin Dean


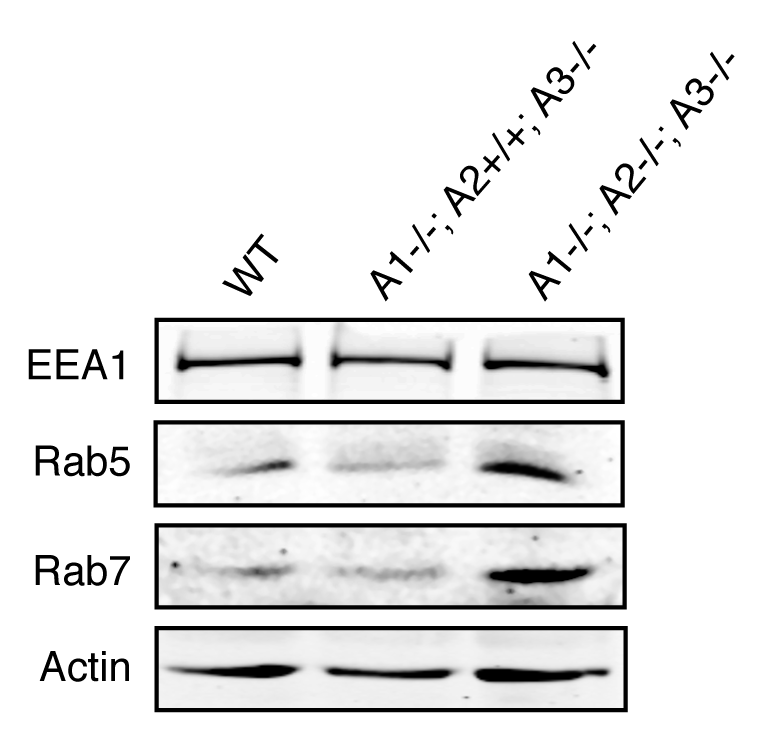


**Supplemental Figure 1.** C: Western blots showing entire blots and levels of EEA1, Rab5 and Rab7 (and Actin as a loading control) in wild-type, EndophilinA1/A3 DKO and EndophilinA TKO brain homogenates.


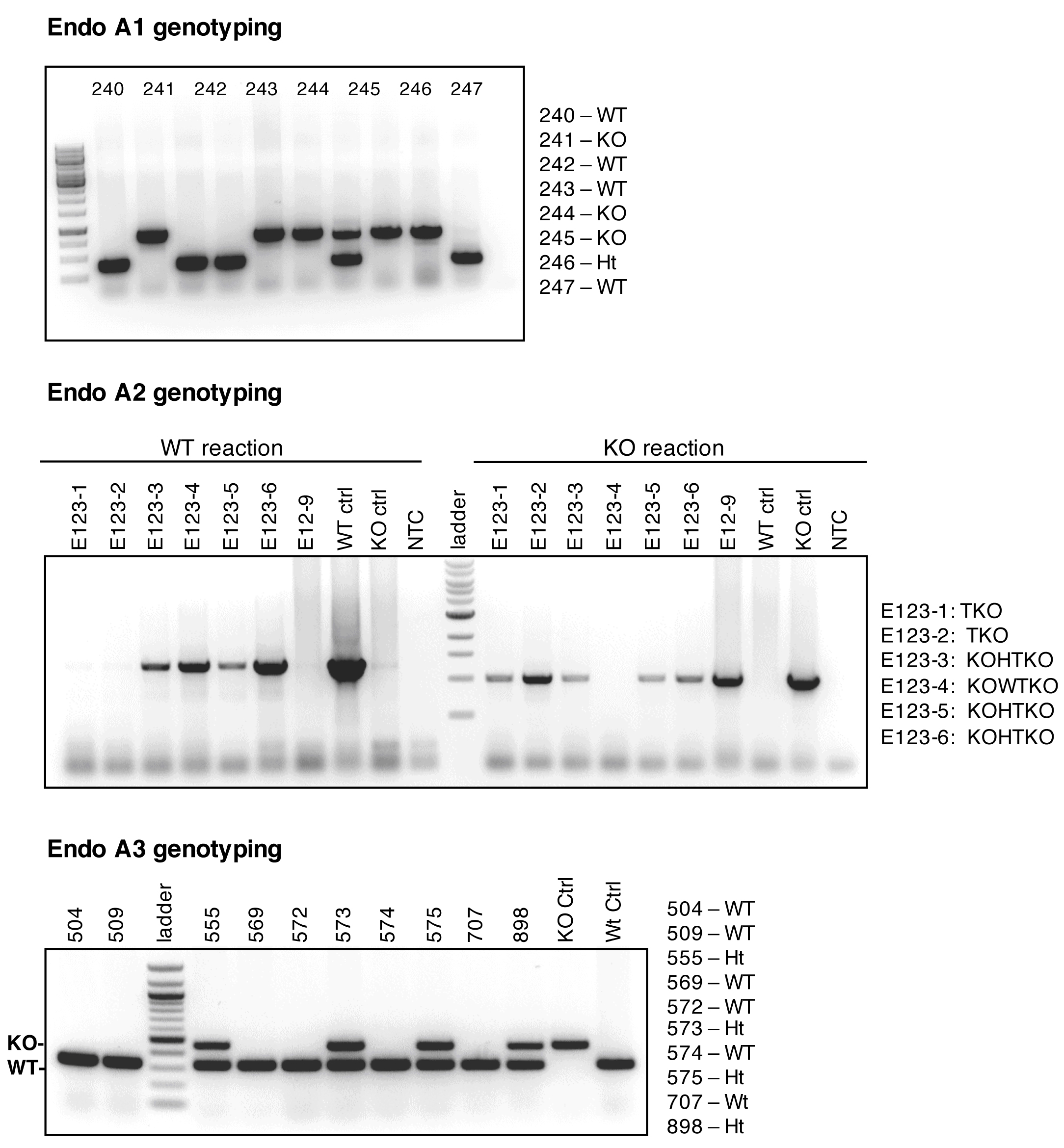


**Supplemental Figure 2**. Examples of genotyping of EndophillinA1, A2, A3 knockout mice.

**Supplemental Movie S1**. Timelapse of EndophilinA2 (green) with Rab7-RFP (red) in the absence of BDNF. Frame interval= 5s.

**Supplemental Movie S2**. Timelapse of EndophilinA2 (green) with Rab7-RFP (red) in the presence of BDNF. Frame interval= 5s.

**Supplemental Movie S3**. Timelapse of EndophilinA2 (green) with Lamp1-RFP (red) in the absence of BDNF. Frame interval= 5s.

**Supplemental Movie S4**. Timelapse of EndophilinA2 (green) with Lamp1-RFP (red) in the presence of BDNF. Frame interval= 5s.

**Supplemental Movie S5**. Timelapse of EndophilinA3 (green) with Rab7-RFP (red) in the absence of BDNF. Frame interval= 5s.

**Supplemental Movie S6**. Timelapse of EndophilinA3 (green) with Rab7-RFP (red) in the presence of BDNF. Frame interval= 5s.

**Supplemental Movie S7**. Timelapse of EndophilinA3 (green) with Lamp1-RFP (red) in the absence of BDNF. Frame interval= 5s.

**Supplemental Movie S8**. Timelapse of EndophilinA3 (green) with Lamp1-RFP (red) in the presence of BDNF. Frame interval= 5s.
